# The Gastrointestinal Helminth *Heligmosomoides bakeri* Suppresses Inflammation in a Model of Contact Hypersensitivity

**DOI:** 10.3389/fimmu.2020.00950

**Published:** 2020-05-19

**Authors:** Kara J. Filbey, Palak H. Mehta, Kimberley J. Meijlink, Christophe Pellefigues, Alfonso J. Schmidt, Graham Le Gros

**Affiliations:** Malaghan Institute of Medical Research, Wellington, New Zealand

**Keywords:** helminth, immunoregulation, skin, infection, contact hypersensitivity (CHS)

## Abstract

Helminths regulate host immune responses to ensure their own long-term survival. Numerous studies have demonstrated that these helminth-induced regulatory mechanisms can also limit host inflammatory responses in several disease models. We used the *Heligmosomoides bakeri (Hb)* infection model (also known as *H. polygyrus* or *H. polygyrus bakeri* in the literature) to test whether such immune regulation affects skin inflammatory responses induced by the model contact sensitiser dibutyl phthalate fluorescein isothiocynate (DBP-FITC). Skin lysates from DBP-FITC-sensitized, *Hb*-infected mice produced less neutrophil specific chemokines and had significantly reduced levels of skin thickening and cellular inflammatory responses in tissue and draining lymph nodes (LNs) compared to uninfected mice. *Hb*-induced suppression did not appear to be mediated by regulatory T cells, nor was it due to impaired dendritic cell (DC) activity. Mice cleared of infection remained unresponsive to DBP-FITC sensitization indicating that suppression was not via the secretion of *Hb*-derived short-lived regulatory molecules, although long-term effects on cells cannot be ruled out. Importantly, similar helminth-induced suppression of inflammation was also seen in the draining LN after intradermal injection of the ubiquitous allergen house dust mite (HDM). These findings demonstrate that *Hb* infection attenuates skin inflammatory responses by suppressing chemokine production and recruitment of innate cells. These findings further contribute to the growing body of evidence that helminth infection can modulate inflammatory and allergic responses via a number of mechanisms with potential to be exploited in therapeutic and preventative strategies in the future.

## Introduction

Various epidemiological studies have drawn a link between the incidence of helminth infection and reduction in allergic and inflammatory diseases (1–4). In seeking mechanisms by which this might occur, experimental models have linked helminth-induced regulatory T and B cells (Tregs and Bregs, respectively), to the dampening of responses to allergens (5, 6) such as HDM (7, 8), ovalbumin (OVA) (9, 10) and peanut (11), and to the amelioration of inflammation in a number of disease models including colitis (12–14), experimental autoimmune encephalitis (15), and diabetes (16, 17).

More recently, mechanisms other than the induction of regulatory lymphocytes have been uncovered that may play a role in protection by helminths. A protein made by *Hb*, a rodent gastrointestinal helminth, was found to actively block the release of IL-33 from necrotic airway epithelial cells after exposure to the fungal allergen *Alternaria*, thus dampening type 2 innate lymphoid cell (ILC2) and subsequent Th2 responses (18). Macrophages from *Trichinella spiralis*-infected donors, or those treated with *T. spiralis* excretory-secretory (ES) products *in vitro*, were found to be protective in models of colitis and OVA-induced allergic airway inflammation when transferred into recipient mice (19). A defense peptide from *Fasciola hepatica* can inhibit local osteoclast formation and limit bone destruction in a model of arthritis (20) and injection of *Schistosoma mansoni* eggs can limit allergic airway inflammation by reducing the influx of monocyte-derived dendritic cells (moDCs) into lung tissue (21). Also, chronic infection with *Litomosoides sigmodontis* protects mice from OVA-induced anaphylaxis by decreasing numbers and activity of mast cells (22).

These observations have given rise to the proposal that elimination of helminth infections from human communities might be linked to the global increase of allergic and atopic skin diseases (23–25). However, there are relatively few pre-clinical studies relating helminth infection in mice to immune responses in the skin, in particular in the context of allergy or contact hypersensitivity (CHS). Therefore, we undertook experiments to investigate the influence of infection with *Hb* on the outcome of a model of skin inflammation in mice. Chronic *Hb* infection is a well-characterized model where the parasite is confined to the small intestine throughout its lifecycle in the host. As well as stimulating a strong Th2 immune response, survival of adult *Hb* in the host intestine appears to be maintained by the expansion of both Tregs and Bregs, induced via various mechanisms including the expression of a TGF-beta molecular mimic in its ES products (26) and the induction of a subset of regulatory DCs that preferentially expand Tregs over Th2 cells (27).

To induce a relevant skin CHS reaction in mice we used a topically applied DBP-FITC prime-challenge model which stimulates a potent neutrophil and Th2 mediated inflammatory response (28–30). DBP is a phthalate ester commonly used as a plasticiser in many cosmetic and industrial products, which acts as an adjuvant to the hapten FITC in models of CHS (31). Inflammation in this model is dependent on type 2 CD4+ T cell responses (32) which are primed by specific subsets of DCs (29, 30, 33) in the local lymph node. The cytokine thymic stromal lymphopoietin (TSLP) has been shown to be key in the maturation and accumulation of these DCs and the subsequent initiation of a Th2 response to DBP-FITC (28, 29).

Strikingly, we found DBP-FITC-induced skin inflammation to be significantly reduced in helminth-infected mice, alongside attenuated accumulation of innate effector cells and a significant reduction in expression of inflammatory chemokines and cytokines in the skin during both the DBP-FITC sensitization and challenge phases of the inflammatory response.

Here, we expand the findings on the therapeutic potential of helminths in the context of models of allergic and inflammatory diseases of the skin and provide a basis for future experiments to investigate more in-depth mechanistic explanations for this phenomenon.

## Materials and Methods

### Mice

6-10-week-old female C57BL/6 mice were used throughout and were bred and maintained by the MIMR Biomedical Research Unit. All procedures were approved by Victoria University of Wellington. Anaesthetisations involved intraperitoneal (i.p.) injection of ketamine/xylazine.

### Parasites

*Hb*, previously identified as *H. polygyrus*, was maintained as previously described (34). *Hb* infection was cleared with two subcutaneous doses of ivermectin (200 μg) (Noromectin, Norbrook Laboratories Ltd, Newry, Northern Ireland) on days 13, 14 of infection. Clearance was confirmed by checking fecal samples for eggs 7 days later, before sensitization with DBP-FITC.

### Contact Sensitization With DBP-FITC

**Figure 1:**
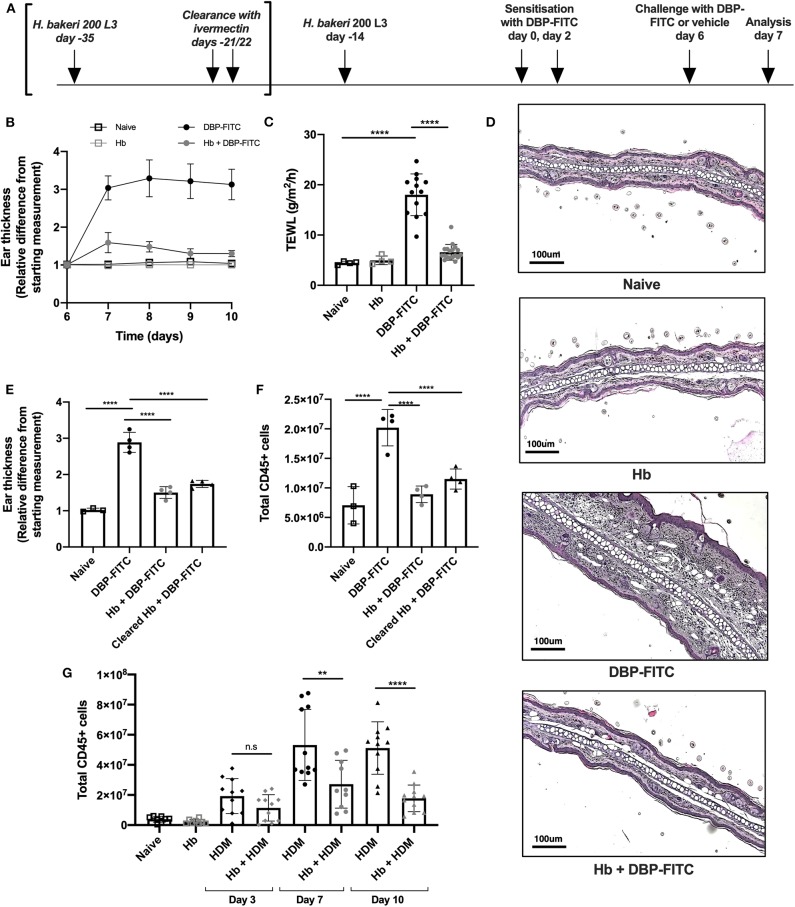
DBP-FITC-induced skin inflammation is suppressed by *H. bakeri* infection. **(A)** Timeline of *Hb* infection and DBP-FITC topical sensitization and challenge model. lvermectin treatment for *Hb* clearance experiments [results in **(E,F)**] is shown in brackets. **(B)** Relative difference in ear thickness compared to day 6 baseline measurements, in naïve mice (black lined clear squares), *Hb*-infected mice (gray lined clear squares) or in mice challenged DBP-FITC (gray circles) or without (black circles) prior *Hb* infection. **(C)** TEWL (g/m2/h) at site of DBP-FITC challenge measured at day 7, or in control naive mice. **(D)** Ear sections stained with H&E on day 7. Scale bars represent 100 um. **(E)** Relative difference in ear thickness in naïve mice, or at day 7 after DBP-FITC challenge in mice with *Hb* infection, or after clearance of the infection. **(F)** Total number of live CD45+ cells in ear dLN at day 7 in the same groups as **(E)**. **(G)** Total number of live CD45+ cells in ear dLN at days 3, 7, and 10 post-HDM intradermal injection into the ear, with or without prior *Hb* infection, or in naive mice. A one-way ANOVA with Tuckey's multiple comparisons test was used to test statistical significance. Graphs represent data combined from 2 experiments **(B,C,G)** or are representative of 2 repeat experiments **(D,E,F)** and show mean ± SD. n.s, not significant; **p < 0.01, ****p < 0.0001.

**Figure 2:**
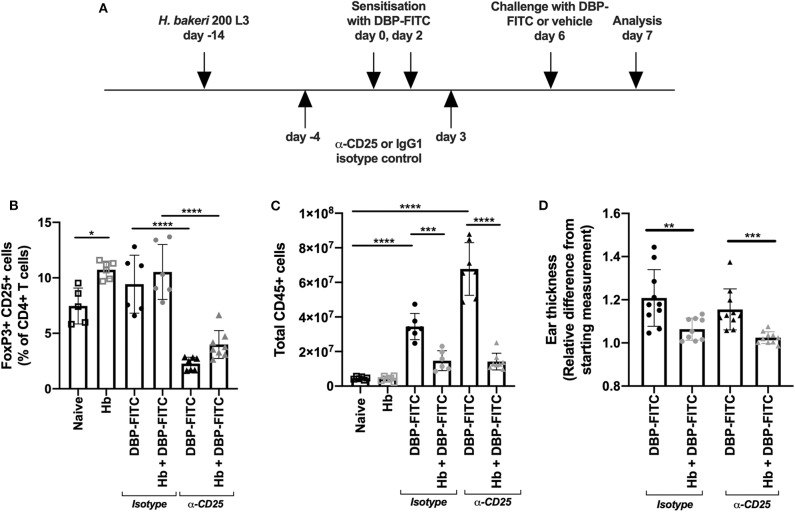
Anti-CD25 depletion of *H. bakeri-* induced Tregs does not affect reduction of LN hyperplasia after DBP-FITC challenge. **(A)** Timeline of DBP-FITC topical application model including treatment with anti-CD25 or isotype control. **(B)** Frequency of FoxP3+ CD25+ cells within the CD4+ T cell compartment at day 7 in the ear dLN. **(C)** Total number of live CD45+ cells from the ear dLN at day 7. **(D)** Relative difference in ear thickness at day 7 after DBP-FITC challenge in mice with or without *Hb* infection, treated with anti-CD25 or an isotype control. A one-way ANOVA with Tuckey's multiple comparisons test was used to test statistical significance. Graphs represent data combined from 2 experiments and show mean ± SD. n.s, not significant; *p < 0.05, **p < 0.01, ***p < 0.001, ****p < 0.0001.

For the sensitization, challenge model (Figure 1A), mice were sensitized on abdominal skin, that had been shaved a week prior, on days 0 and 2 by epicutaneous application of 40 μl 0.5% FITC (Sigma) in 1:1 mix of DBP (Aldrich) and acetone (Sharian). At day 6, a challenge dose of 20 μl DBP-FITC was applied to one ear pinna and 20 μl of vehicle (DBP only) to the other. For assessment of DC migration to the ear-draining LNs, mice were treated with 20 μl DBP-FITC on both ears at day 0 and LNs harvested on day 2. For the Treg depletion experiments, mice were treated twice with DBP-FITC, on days 0 and 2 (see Figure 2A), as was the case for assessment of cells in the ear tissue. For assessment of cytokines and chemokines in the ear tissue, mice were treated once with DBP-FITC on each ear and tissue was harvested at the timepoints indicated. Ear thickness was measured with a digital micro-meter (Micromaster, Capa System) and is presented as fold-change from the starting measurement on day 6, measured separately for each group. Trans-epidermal water loss (TEWL) was measured with a TEWL open chamber unit (Dermalab). Naïve control mice were neither infected or treated with any compound.

### Injection of HDM

Mice were anesthetized and intradermally injected with 200 μg crushed *Dermatophagoides pteronyssinus* (Greer) in 30 μl sterile PBS as previously described (35).

### Treg Depletion

Mice were injected i.p. with 500 mg anti-CD25 antibody (PC61; BioXCell) or rat IgG1 isotype control (BioXCell) in sterile PBS, at the time points indicated in Figure 2A.

### Tissue Digestion and Flow Cytometry

**Figure 3:**
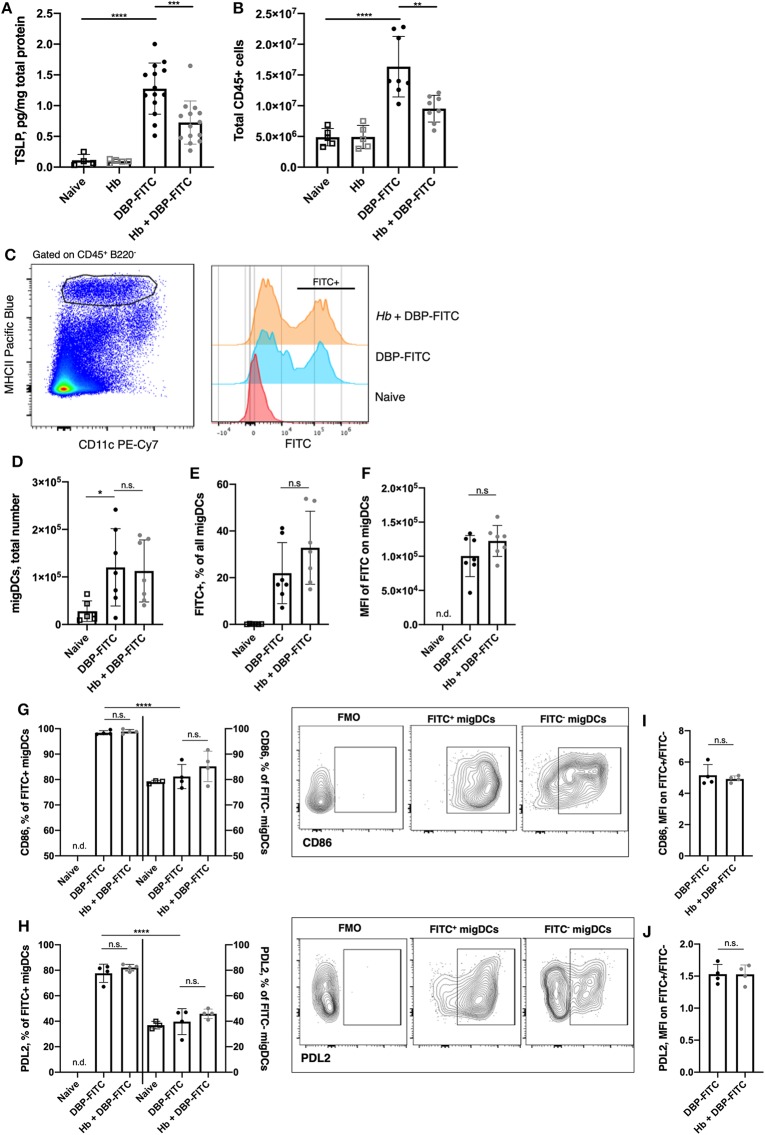
*H. bakeri* infection does not affect DC uptake and transport of DBP-FITC to the skin draining LN after sensitization. **(A)** TSLP protein levels 24 h post-sensitization with DBP-FITC in tissue lysate measured by ELISA. **(B)** Total number of live CD45+ cells in the ear dLN at 2 days post DBP-FITC sensitization. **(C)** Representative plots of FITC expression by CD45^+^ B220^−^ CD11c^var^ MHCII^high^ migratory DCs (migDCs) in the ear dLN at day 2. **(D)** Total number of migratory DCs (migDCs) in the ear dLN at day 2. **(E)** Frequency of FITC+ migDCs in ear dLN at day 2. **(F)** Median fluorescence intensity (MFI) of FITC on migDCs in the ear dLN at day 2. **(G)** Frequency of FITC^+^ and FITC^−^ DCs expressing CD86 or **(H)** PDL2 in the ear dLN at d2, with plots showing representative gating. **(I)** Ratio of MFI of CD86 or **(J)** PDL2 expression in FITC+ vs. FITC- migDCs in the dLN at d2. A one-way ANOVA with Tuckey's multiple comparisons test was used to test statistical significance between multiple groups **(A–H)**, or an unpaired *t*-test between 2 groups **(I,J)**. FMO, fluorescence minus one. Graphs represent data combined from 2 experiments **(A–F)** or are representative of 2 repeat experiments **(G–J)**. Graphs show mean ± SD. n.s, not significant; *p < 0.05, **p < 0.01, ***p < 0.001, ****p < 0.0001. n.d, not detectable.

LNs were dissociated and passed through a 70 μm strainer before enumeration. For DC analysis LNs were teased apart followed by digestion in 100 μg/mL DNase I (Sigma) and 100 μg/mL Liberase TL (Roche, Germany) for 25 min at 37°C and passed through a 70 μm strainer. For skin cell preparations, ears were split into the dorsal and ventral layers, minced and digested for 30 min, shaking at 150 rpm in a 37°C incubator, in IMDM (Gibco) plus 5% FCS (Gibco) containing 2 mg/ml collagenase IV and 100 μg/mL DNase 1 (both Sigma). Digestion was stopped by adding 10% FBS plus 5 mM EDTA in HBSS and a single cell suspension was obtained by passing through a 70 μm strainer. Skin single cell suspensions and LN cells were stained for surface markers as previously described (36). Viability was assessed by staining with Live/Dead Fixable Blue Viability stain (Invitrogen), Zombie NIR (Biolegend) or DAPI (Sigma). Antibodies used were as follows: MHCII-Pacific Blue (M5/114.15.2), CD3-PE-Cy5 (145-2C11), CD11b-BV570 (M1/70), CD4-BV750 (GK1.5), CD64-PE-Dazzle594 (X54-5/7.1), Ly6C-AF700 (HK1.4), CD45-APC-Cy5.5 (104, all from Biolegend), B220-PE-CF594 (RA3-6B2), CD45-BUV395 (30.F11), CD3-BV786 (145-2C11), CD4-BV605 (RM4-5), CD8a-PE-Cy7 (53-6.7), CD25-BV421 (7D4), CD3-BV711 (145-2C11), CD11c-PE-Cy7 (HL3), CD11b-BUV395 (M1/70), CD326-BV711 (G8.8), CD86-PE (GL1), SiglecF-BV421 (E50-2440), Ly6G-BV711 (1A8, all from BD), and CD103-APC (E2E7 from eBioscience). Migratory DCs were characterized as CD45^+^ B220^−^ CD11c^var^ and MHCII^high^ as previously described (29)—see Figure 3C. For intra-nuclear staining the FoxP3-PE/Transcription Factor Staining Buffer set (eBioscience) was used as per manufacturer's instructions. Regulatory T cells were defined as CD4^+^ FoxP3^+^ CD25^+^. All gating was based on fluorescence minus one (FMO) controls where applicable. Data were acquired using a BD LSRII or Cytek Aurora, and analyzed using FlowJo v10.

### Sectioning, Staining, and Microscopy

For haematoxylin and eosin (H&E) staining, whole ears were fixed in 10% formalin (Sigma) for 24 h and dehydrated into xylene (Pronalys) overnight in a tissue processor (Thermo Fisher Citadel 200). Paraffin wax embedded tissue was sectioned on a microtome at 3–4 μm onto adhesive microscope slides (Trajan). Sections were rehydrated and stained with hematoxylin and eosin as per manufacturer's instructions (Thermo Fisher). Sections were observed using an Olympus BX51TF compound microscope using a 10x, N.A. 0.3 objective, and images taken in the middle of the ear section.

For confocal microscopy, samples were processed and stained using a standard immunofluorescence protocol (37). Whole ears were incubated in 20% sucrose for 1 h and snap-frozen in OCT compound (Tissue-Tek) using a Stand-Alone Gentle Jane snap-freezing system (Leica Biosystems). Cryosections of 10 μm were fixed in cold acetone for 3 min and blocked with Fc Block (clone 2.4G2) for 1 h and stained with Ly6G-PE (clone 1A8, Pharmingen). For nuclear staining, sections were incubated with DAPI (2 mg/ml) for 10 min. Images were taken with an inverted IX 83 inverted microscope equipped with a FV1200 confocal head (Olympus) using a 20X, N.A. 0.75 objective. Images were acquired using the FV10-ASW software (v4.2b, Olympus) and processed with ImageJ (38).

### Cytokine and Chemokine Quantification in Skin Tissue

Ear tissue was finely chopped into 1x Lysis Buffer (Cell Signaling Technology Inc.) with 1:100 phenylmethanesulfonyl fluoride solution (PMSF) (Sigma) and homogenized with 5 mm stainless steel beads (Qiagen) using a TissueLyserII (Qiagen). Samples were centrifuged and cytokines and chemokines measured in the supernatant with LEGENDplex multi-analyte assay kits (Biolegend). TSLP was measured with an ELISA DuoSet (R&D Systems). Results were normalized to total protein content in the sample measured by Bradford assay (Thermo Fisher).

### Statistical Analysis

All statistical analyses were performed using Prism v8 (GraphPad). An unpaired *t*-test was used to compare two groups, and a one-way ANOVA with Tuckey's multiple comparisons test with adjusted *p*-values was used to compare several groups. Tests used are indicated in each figure legend. Significant results are indicated as follows: ^*^ = *p* < 0.05, ^**^ = *p* < 0.01, ^***^ = *p* < 0.001, ^****^ = *p* < 0.0001. n.s. = not significant. All symbols represent individual samples and error bars represent mean ± standard deviation (SD).

## Results

### DBP-FITC-Induced Skin Inflammation and LN Hyperplasia Are Suppressed by *H. bakeri* Infection

Topical application of DBP-FITC to the ear skin of mice induces Th2-dominated CHS inflammatory responses with similar characteristics to human atopic skin disease or atopic dermatitis (AD) (28, 32). We sought to determine the effect of chronic helminth infection on skin inflammatory responses by infecting mice with *Hb* 14 days prior to sensitization with DBP-FITC. At this time point in infection adult *Hb* are present in the lumen of the small intestine of most host mouse strains (including C57BL/6), and are known to stimulate a strongly polarized type 2 response (39). Both *Hb*-infected and control mice were sensitized to DBP-FITC on day 0 and day 2 with topical applications to the abdomen and then challenged with one DBP-FITC application to both sides of the ear at day 6. Analysis of ear tissues was completed 24 h after challenge corresponding to day 7 of the timeline (Figure 1A). Skin inflammation, as measured by swelling of the ear dermis, peaked 24 h after the day 6 challenge with DBP-FITC, and slowly reduced in size thereafter (Figure 1B). Trans-epidermal water loss (TEWL), a surrogate marker for the loss of skin barrier function at the site of DBP-FITC challenge (40), increased significantly 24 h after the day 6 challenge (Figure 1C). By comparison, *Hb-*infected mice had similar TEWL levels at the site of DBP-FITC challenge, compared to naive mice (Figure 1C). Haematoxylin and eosin (H&E) staining revealed a significant inflammatory infiltrate in the ear dermis following the challenge application of DBP-FITC, which was completely absent in *Hb*-infected mice (Figure 1D). To determine whether the effect of *Hb* infection on the DBP-FITC-induced inflammatory response was dependent on the presence of live worms secreting biologically active molecules, we used ivermectin to clear mice of adult worms and measured the subsequent DBP-FITC-induced skin inflammation and LN hyperplasia. Strikingly, mice cleared of *Hb* continued to benefit from reduced skin inflammatory responses (Figure 1E) and diminished LN hyperplasia (Figure 1F). Taken together, these data indicated that it was not the immediate action of short-lived regulatory molecules secreted by the live parasite that was diminishing the inflammatory responses to DBP-FITC.

Injection of house dust mite (HDM) intradermally into the ear results in a rapid and robust Th2 response in the draining auricular LN (35). In accordance with our findings with DBP-FITC, prior *Hb* infection also limits inflammation in the dLN after HDM injection at all time points measured (Figure 1G).

### Anti-CD25 Depletion of *H. bakeri*-Induced Tregs Does Not Affect Reduction of Skin Inflammation

*Hb* is known to induce CD4^+^ FoxP3^+^ CD25^+^ T regulatory (Treg) cells in local lymphoid organs, and these cells represent a key feature of the helminths ability to limit organ specific inflammation in a variety of disease models (41). We investigated whether the *Hb-*dependent suppression of DBP-FITC-induced inflammation and skin draining LN hyperplasia could be neutralized by a Treg specific monoclonal antibody depletion regime (Figure 2A). We found that helminth infection modestly increased the frequency of FoxP3^+^ CD25^+^ Tregs within the CD4^+^ T cell compartment in the auricular LN draining the ear skin, and that 2 doses of anti-CD25 significantly reduced the frequency of Tregs in both uninfected and *Hb* infected mice (Figure 2B). Depletion of Tregs in DBP-FITC treated mice increased LN hyperplasia, illustrating that Tregs play some role in muting inflammatory responses even without the influence of a helminth infection. However, Treg depletion did not affect the *Hb*-induced suppression of the LN response to DBP-FITC (Figure 2C) or the reduction in skin thickness measured 24 h after challenge (Figure 2D). Taken together these experiments indicate that Tregs do not appear to play a major role in *Hb-*induced suppression of the DBP-FITC inflammatory response.

### *H. bakeri* Infection Does Not Affect the DC-Mediated Uptake and Transport of DBP-FITC to the Skin Draining LN

The DBP-FITC model of CHS is dependent on the expression of TSLP, which acts on DCs to induce Th2 responses in the skin and local LN (28, 29, 42, 43). As expected, TSLP production in the skin was increased 24 h following DBP-FITC skin sensitization (Figure 3A). We found that prior *Hb* infection leads to a significantly decreased amount of TSLP being detected in the skin lysate.

We used the FITC signal detected on skin DCs to track allergen uptake, transport to the draining LN and expression of activation markers to determine whether *Hb* infection had any effect on DC function and immune responses in the draining auricular LN (29, 30). We found that 2 days after DBP-FITC application to the ear skin of naive mice there was significant LN hyperplasia detected (measured by quantifying live CD45^+^ cells in the LN) and this was curtailed in *Hb*-infected mice (Figure 3B). The number of migratory DCs (defined as CD45^+^ B220^−^ CD11c^var^ MHCII^high^, Figure 3C) detected by flow cytometry in the skin dLN was not affected by *Hb* infection (Figure 3D). Neither was the proportion of these that were positive for FITC (Figure 3E), and the level of FITC uptake (illustrated by the median fluorescent intensity of the FITC detected) was the same in *Hb* and uninfected mice (Figure 3F). Furthermore, uptake of the FITC antigen leads to almost ubiquitous expression of CD86 compared to the lower proportion of FITC^−^ DCs that express this activation marker (Figure 3G). Of note, *Hb* infection does not alter these proportions in either FITC^+^ or FITC^−^ DCs. The degree of CD86 expression by FITC^+^ compared to FITC^−^ DCs is around 5-fold higher (Figure 3I) and this ratio is not affected by *Hb* infection. Programmed death ligand-2 (PDL2), is a marker expressed on a subset of DCs that promote Th2 responses *in vitro* and *in vivo*, and are enriched in skin dLN (44). PDL2 was upregulated on FITC^+^ compared to FITC^−^ DCs in the LN after DBP-FITC sensitization, but again, *Hb* infection did not have an impact on the proportion of DCs expressing this marker (Figure 3H). Expression of PDL2 is around 1.5-fold higher on FITC^+^ DCs and this is not altered by prior *Hb* infection (Figure 3J).

Collectively, these experiments show that although *Hb* infection reduced the levels of TSLP that could be detected in the skin, it did not appear to be enough of a reduction to translate into defects in migration to, or activation of DC in, the skin dLN.

### Expression of Skin Derived Inflammatory Chemokines and Influx of Innate Cells Are Reduced in *H. bakeri* Infected Mice

**Figure 4:**
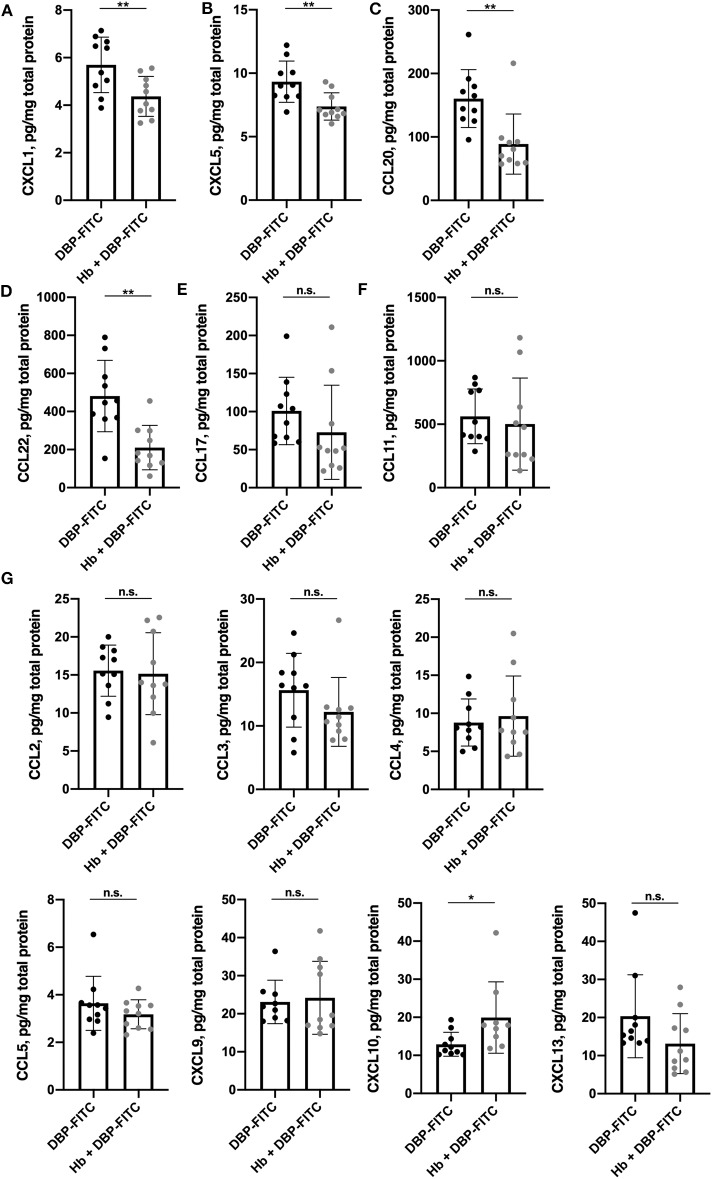
Expression of a subset of skin-derived inflammatory chemokines is reduced in *H. bakeri*-infected mice after DBP-FITC sensitization. Chemokine protein expression in tissue lysate measured by LEGENDplex 12 h post- sensitization with DBP-FITC. **(A)** CXCL1, **(B)** CXCL5, **(C)** CCL20, **(D)** CCL22, **(E)** CCL17, **(F)** CCL11, **(G)** CCL2, 3, 4, and 5 and CXCL9, 10, and 13. Results are expressed as pg/mg total protein measured by Bradford assay. An unpaired *t*-test was used to assess statistical differences. Graphs represent data combined from 2 experiments and show mean ± SD. n.s., not significant, *p < 0.05, **p < 0.01.

We next considered whether *Hb* infection affected the local production of chemokines in the skin. There is a complex profile of chemokines expressed in skin after exposure to contact sensitisers, which orchestrates the influx of inflammatory cells throughout the course of the immune response (45). Skin tissue lysates were analyzed 12 h after DBP-FITC sensitization for a range of widely recognized inflammatory chemokines using a 13-plex bead array. Strikingly, the levels of the pro-inflammatory chemokines CXCL1 (or keratinocyte chemoattractant; KC) and CXCL5 (lipopolysaccharide-induced CXC chemokine; LIX) were found to be significantly reduced in the skin lysates of *Hb*-infected mice sensitized 12 h previously with DBP-FITC when compared to the skin lysates of uninfected mice sensitized with DBP-FITC (Figures 4A,B). Keratinocytes in the skin produce CXCL1 to recruit neutrophils in the initial stages of CHS (46), and CXCL5 is a neutrophil chemo-attractant in several skin inflammation settings (47, 48). Protein levels of CCL20 (macrophage inflammatory protein-3; MIP-3-alpha) and CCL22 (macrophage-derived chemokine; MDC) were also significantly reduced in skin from *Hb*-infected mice (Figures 4C,D). CCL20 acts to recruit immature DCs, effector and memory T cells, and to a lesser extent neutrophils, to sites of skin inflammation via interaction with its receptor CCR6 (49), and has been linked to inflammation of the skin in the context of psoriasis and AD in mice and humans (50–52). CCL22 plays a key role in the accumulation of CD4^+^ T cells in the skin in AD and CHS (53, 54). Expression of the T cell recruiting chemokine CCL17 (thymus and activation regulated chemokine; TARC) was also reduced in *Hb*-infected mice, although this change did not reach statistical significance (Figure 4E).

CCL11 (eotaxin), the main chemoattractant for eosinophils, was detected in DBP-FITC treated skin but there was no difference in amounts detected between the lysates from control and *Hb*-infected mice (Figure 4F). None of the other chemokines measured were found to be downregulated by *Hb* infection (Figure 4G). Of note, skin left untreated in naïve mice produces very low levels of the inflammatory chemokines mentioned above, as application of the contact sensitiser is needed to elicit these early inflammatory events (45).

**Figure 5:**
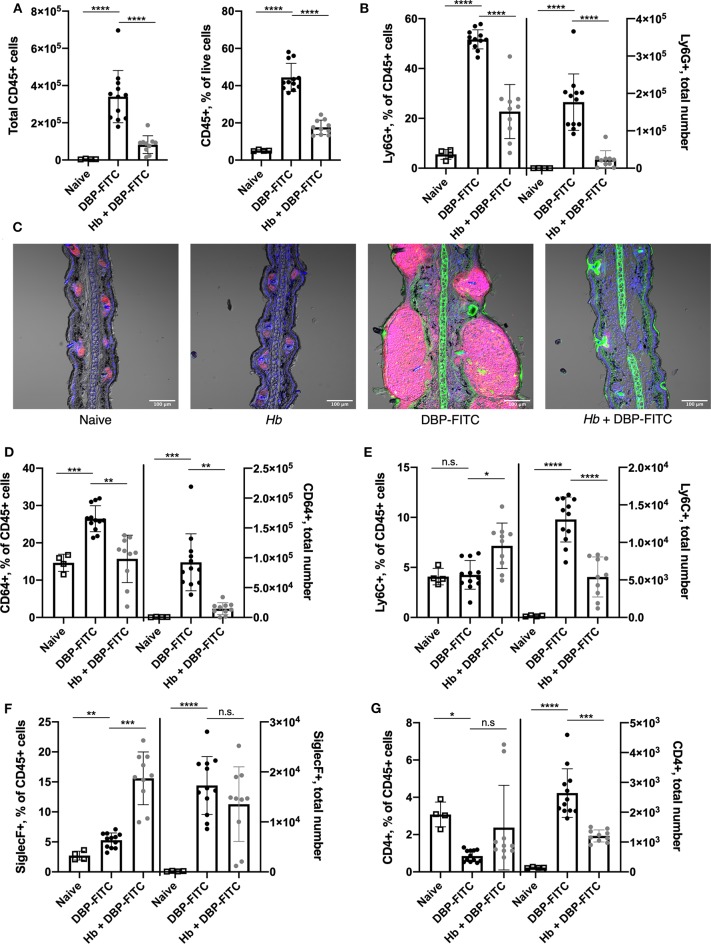
Prior Hb infection limits influx of innate inflammatory cells after DBP-FITC challenge. **(A)** Total number of CD45+ cells and their frequency within all live cells in the skin tissue at day 7 (24 h after DBP-FITC challenge). **(B)** Frequency within CD45+ cells, and total number of neutrophils (Ly6G+) in the skin at day 7. **(C)**. Immunofluorescent staining of ear sections taken at day 7. Ly6G+ neutrophils (pink), FITC (green) and DAPI (blue). Scale bar represents 100 um. **(D)** Frequency within CD45+ cells and total number of macrophages (CD64+), **(E)** monocytes (Ly6C+), **(F)** eosinophils (SiglecF+), and **(G)** CD4+ T cells in the skin at day 7. A one-way ANOVA with Tuckey's multiple comparisons test was used to test statistical significance. Graphs represent data combined from 2 experiments and show mean ± SD. n.s, not significant; *p < 0.05, **p < 0.01, ***p < 0.001, ****p < 0.0001.

Since a number of key chemoattractant molecules were down-regulated in the skin of *Hb*-infected mice, we next determined whether the subsequent recruitment of innate and adaptive immune cell populations to the skin 24 h after DBP-FITC challenge was affected. Indeed, DBP-FITC challenge leads to a robust recruitment of CD45^+^ cells to the ear skin tissue shown by both the absolute numbers and the proportions of live cells expressing CD45 (Figure 5A). In contrast, *Hb*-infected mice recruit significantly fewer CD45^+^ cells when challenged with DBP-FITC. Even more strikingly, and corresponding with the decrease in production of the chemokines CXCL1 and CXCL5, proportions and total numbers of Ly6G^+^ neutrophils within the CD45^+^ cell compartment were significantly reduced in the ears of DBP-FITC-treated mice infected with *Hb* (Figure 5B). This finding was confirmed by immunofluorescent staining of ear sections which showed that after DBP-FITC challenge, a robust influx of neutrophils causes swelling and structural disruption under the skin surface (Figure 5C). Corroborating the flow cytometry results in Figure 5B, *Hb* infection almost completely abrogates this neutrophilic inflammation. Neutrophils are key innate effector cells in the initiation of CHS, and in their absence, CHS responses cannot be induced (46, 55).

There is a heterogenous population of macrophages, monocytes and moDCs in the skin (47, 48). Interestingly, we observed an increased proportion and number of skin CD64^+^ macrophages in mice treated with DBP-FITC and these were significantly reduced in mice infected with *Hb* (Figure 5D). A similar trend was observed in numbers of Ly6C^+^ monocytes, although a less striking difference is seen in the proportions of these cells (Figure 5E). Eosinophils are a characteristic effector cell involved in allergic skin responses (32, 56) and were found in increased numbers in the skin after DBP-FITC treatment compared to in naïve mice (Figure 5F). Interestingly, *Hb* infection significantly increased the proportions of eosinophils within the CD45^+^ compartment, although this did not translate into a change in numbers found in the skin between uninfected and infected mice treated with DBP-FITC (Figure 5F). This finding correlated with the similar levels of eosinophil chemoattractant CCL11 that were detected in the skin lysate in both uninfected and *Hb*-infected mice (Figure 4F). Skin inflammation in this DBP-FITC CHS model is CD4^+^ T cell-dependent (32) and we found a significant increase in numbers of these cells in the skin after challenge compared to naïve mice which again, was decreased by prior helminth infection (Figure 5G). Of note, innate cells including neutrophils, macrophages, monocytes and eosinophils account for the majority of the inflammatory cellular milieu at the time point assessed.

Taken together, these results suggest that *Hb* infection limits the DBP-FITC-induced accumulation of some key innate inflammatory cell populations in the skin, in particular neutrophils, via a reduced production of the chemokines associated with their recruitment into skin tissues.

## Discussion

We have identified that mice infected with the intestinally confined helminth parasite *Hb* mount impaired inflammatory responses to skin allergens such as DBP-FITC and HDM when compared to uninfected mice. The suppression of skin and draining LN responses to DBP-FITC appeared to be due to a reduction in the release of chemokines involved in attracting neutrophils to sites of inflammation. Neutrophils are the first cells to respond to infection or damage in many tissues (57). Depletion of neutrophils, using either a regime of monoclonal antibody injection (46) or via genetic deficiency (55), has illustrated how important these cells are in both the sensitization and challenge phases of CHS. Helminth infection has previously been shown to limit skin inflammation via the downregulation of chemokines that attract neutrophils (58). However, other molecules such as the eicosanoid leukotriene B4 (59) and the transcription factor NF-E2–related factor-2 (Nrf2) (60) play critical roles in the recruitment of neutrophils to the skin during inflammation. Future work could assess whether molecules in the skin other than chemokines are affected by helminth infection, and whether depletion of neutrophils can indeed mimic the effects of *Hb* in our particular model of CHS.

Although we also saw a significant reduction in numbers of macrophages and monocytes in the skin of *Hb*-infected mice 24 h after DBP-FITC challenge, this did not correlate with reduced expression of chemokines normally associated with these cells earlier in the model (Figure 4). This may be due to the fact that chemokines are known to be sequentially expressed in skin during CHS (61) and so undertaking a more thorough examination of chemokine expression over a time-course of inflammation may reveal differences between infected and uninfected mice. Also, we did not stain for chemokine receptors on the surface of the cells from the skin, so it may also be that although chemokine levels themselves are similar in uninfected and *Hb*-infected mice, helminth infection could impact the cells' responsiveness, due to decreased receptor expression. *In vitro* experiments testing the ability of different cell types from uninfected and infected donors to migrate along a chemokine gradient, or *in vivo* cell tracking experiments may shed light on the mechanisms involved.

Proportions of eosinophils are increased in the skin in CHS as we (Figure 5F) and others have shown (28, 32). However, although *Hb* infection increased the proportions of SiglecF^+^ cells within the CD45^+^ compartment in the skin, this did not translate to absolute numbers of eosinophils counted (Figure 5F). Perhaps surprisingly, we saw no differences between infected and uninfected mice in early expression of the eosinophil chemoattractant CCL11 after DBP-FITC challenge, and so measurement of other molecules that recruit and activate eosinophils, such as IL-5 would be logical. Similar results have been noted in a previous study that found chronic *Litomosoides sigmodontis* infection protected against skin hypersensitivity (58). In this study, eosinophil numbers in the skin after allergen challenge were not affected by helminth infection, however, their production of the neutrophil chemoattractants CXCL1 and CXCL2 were, and this resulted in a greatly reduced neutrophil influx (58). Therefore, it is a possibility the functionality of eosinophils could also be affected by *Hb* infection in our model and further experiments would be needed to investigate this.

Several papers have demonstrated that mast cells are important for CHS responses and that they play a role in recruitment of neutrophils to the skin via production of chemokines (55, 62). Basophils have also been implicated in allergic skin responses, in particular in IgE-mediated allergic dermatitis models, where allergen-specific IgE mediates the activation and degranulation of basophils to release their inflammatory mediators via ligation of the high affinity IgE-receptor FcER1 on their surface and cross-linking by the allergen molecule (63–65). Interestingly, one paper investigating different types of allergic response found that contact hypersensitivity with a chemical hapten was not affected by depletion of basophils (64), whereas another found a significant attenuation of ear swelling after hapten application in basophil depleted mice (66). Although we did not assess mast cell or basophil recruitment in our current model, previous work from our group has shown, using the Basoph-8 reporter mice (67), that basophils are recruited to the skin after injection with the allergen HDM and after prolonged topical application of a vitamin D analog MC903 that induces AD-like skin inflammation (68), and these mice could be utilized in the future to further understand the role of basophils in the *Hb*/DBP-FITC model. Indeed, studies in both mice and humans have implicated that helminth infection can reduce the responsiveness of basophils to IgE (69, 70). Measuring the production of allergen-specific antibodies, particularly IgE, would be an interesting next step in our model as well as assessment of basophil responsiveness to antibody.

DCs are vital for the initiation of the immune response to haptens in the skin and several studies have dissected the roles for the different DC subsets at this stage. Mice lacking epidermal Langerhan's cells or dermal CD103^+^ DCs can still elicit Th2 responses in the dLN after DBP-FITC sensitization (30), with the highest number of FITC^+^ DCs in the LN made up of IRF4^+^ CD11b^+^ and CD11b^lo^ CD103^lo^ CD326^lo^ CD301b^+^ dermal DCs which express high levels of MHCII and the activation marker CD86, and are involved in other Th2 skin responses (30, 71–73). Signaling through the TSLP receptor via STAT5 in DCs is necessary for the initiation of Th2 responses in the skin draining LN (74) and dermal DC subsets that are preferentially activated in the DBP-FITC CHS model are highly responsive to TSLP (28, 29). We found that expression of TSLP was reduced in the skin of *Hb*-infected mice after DBP-FITC treatment, although this did not appear to affect DC antigen uptake, activation or recruitment to the draining LN during the early stages of the inflammatory response. We did not see a difference in proportions of CD4^+^ T cells in the skin after DBP-FITC challenge in uninfected and infected mice. However, assessment of their activation by DCs, in terms of cytokine output upon restimulation would be a logical experiment to perform in the future.

Although previous work has highlighted the importance of induced Tregs in helminth-mediated suppression of immune responses [reviewed recently in (75)], our work points to a Treg-independent mechanism, as Treg depletion had no discernible effect on *Hb*-induced suppression of DBP-FITC inflammation. However, as the anti-CD25 depletion regime is not 100% effective (our protocol resulted in a 60–75% depletion of CD4^+^ FoxP3^+^ Tregs in the skin draining LN), the effect of helminth-induced Tregs cannot be completely ruled out and more complete models of Treg deletion [such as diptheria toxin injection into FoxP3-iDTR (76) or DEREG mice (77)] could be used in the future to confirm this finding.

Individual molecules isolated from the *Hb* excretory-secretory milieu have been found to have therapeutic potential in a number of inflammatory disease settings (78). However, our results showing a continued suppression of DBP-FITC-induced inflammation in the ear after worm clearance suggest that the active secretion of molecules by live worms into the intestinal environment does not directly impact cellular infiltration into the skin and draining LN after DBP-FITC challenge. However, this does not preclude either a long-lasting effect on cells by helminth-derived molecules, or the secretion of molecules that have long half-lives and long-range effects. *Hb* excretory-secretory products (HES) contain a complex mix of proteins (79, 80) and only a few have been characterized fully (18, 79, 81–83). So far, the half-lives and pharmaco-kinetics of individual, or families of, molecules made by the helminth are yet to be determined. An effect of long-lived and long-range worm-derived molecules, present even after infection is cleared by drug therapy, cannot be ruled out and further experiments could be undertaken to further discriminate the role of HES in this model. Intriguingly, injection of *Schistosoma mansoni* egg-derived chemokine binding protein (SmCKBP) limits neutrophil infiltration in a model of CHS by neutralizing the activity of several neutrophil-specific chemokines (84) and the hookworm *Ancylostoma caninum* produces a glycoprotein that inhibits neutrophil migration *in vitro* (85). Furthermore, analogs of the immunomodulatory molecule ES-62 from *Acanthocheilonema viteae* can ameliorate oxazolone-induced skin inflammation (86). Interestingly, it has been shown that the main cellular targets for glucocorticoids, the widely used anti-inflammatory treatment for allergic skin conditions, are macrophages and neutrophils (the cell types we found to be most impacted in infected mice), via binding of the glucocorticoid receptor (GR) (87). It is possible that *Hb* secretes a molecule that binds to GR on these cells to directly modulate their function or activation and prevent their accumulation into the skin in infected mice after DBP-FITC challenge. Previous studies have shown that other helminths do produce steroid hormones (88) but it is not known whether *Hb* makes such molecules.

One other study utilizing *Hb* infection and skin allergic responses showed that *Hb* did not reduce inflammation after prolonged epicutaneous OVA application, although in concordance with our findings, there was evidence of decreased CD4^+^ T cell infiltration into the skin in infected mice (89). The model used is technically very different from ours, using OVA applied for long periods of time (three, 1-week long applications on skin patches) with severity of disease measured by an observational clinical skin score and cells counted after immunohistological staining (89). Importantly, in this study, mice were infected with *Hb* larvae at the same time as systemic sensitization to the OVA allergen was started. In our model, the larvae had already had 2 weeks to undergo full maturation and were established in the small intestine as adults before the DBP-FITC model was initiated. This may point to a role for adult worms at the early stages of sensitization, or a change in systemic distribution or activation of immune cells by the presence of worm infection that alters the response in distal sites to an allergen upon first encounter. Supporting this, *Hb* has been shown to alter allergic responses in a number of tissues distal to the site of the infection in the intestine, and many of the experimental models use infection before the onset of allergic sensitization (7, 11). It would be interesting to see if infecting mice in between allergen sensitization and challenge in our DBP-FITC model would alter the magnitude of the response compared to in uninfected animals. A model of this kind would also mirror a scenario more likely to arise in human conditions whereby intervention to prevent allergic responses would likely occur after sensitization occurred rather than beforehand.

There is evidence from epidemiological studies showing an inverse correlation between infection with helminths and severity of allergic reactions in the skin in humans (1, 2, 90, 91). Our study points to the interesting possibility that controlled infections of humans with helminths of a similar nature to *Hb* could be used to prevent and potentially treat chronic skin diseases such as atopic dermatitis and psoriasis. The potential for therapeutic treatment with helminths for a number of inflammatory and allergic diseases is already being explored with mixed results (92–95). Additionally, helminth-derived molecules have recently started to be tested in human subjects (96), and could yet be shown to have a role in limiting inflammation in the skin, perhaps upon topical application.

Importantly, we have preliminary evidence that a helminth-induced amelioration of skin responses is not limited to DBP-FITC-induced inflammation, but extends to other clinically relevant skin inflammation models, including the injection of the ubiquitous HDM (Figure 1) and the application of a vitamin D analog contact sensitizer MC903 (manuscript in preparation).

## Data Availability Statement

The datasets generated for this study are available on request to the corresponding author.

## Ethics Statement

The animal study was reviewed and approved by Animal Ethics Committee, Victoria University of Wellington, New Zealand.

## Author Contributions

KF conceptualized the project, performed and supervised the experiments, analyzed and interpreted data, and wrote the manuscript. PM developed methodologies, performed experiments, and edited the manuscript. KM developed methodologies and performed experiments. CP provided expertise, developed methodologies, and performed experiments. AS provided expertise. GL supported design of the project and experiments, supported analysis of the data, and edited the manuscript.

## Conflict of Interest

The authors declare that the research was conducted in the absence of any commercial or financial relationships that could be construed as a potential conflict of interest.
